# Professionals’ ideas and observations on preschoolers’ experiences with physical symptoms: a qualitative interview study

**DOI:** 10.1186/s12887-024-05157-4

**Published:** 2024-11-06

**Authors:** Sterre van der Ziel, Janna M. Gol, Daniël Schoemaker, Judith G. M. Rosmalen, Michel J. van Vliet

**Affiliations:** 1 University of Groningen, University Medical Center Groningen, Department of Psychiatry, PO Box 30001, Groningen, 9700 RB the Netherlands; 2 University of Groningen, University Medical Center Groningen, Department of Internal Medicine, Groningen, the Netherlands; 3 University of Groningen, University Medical Center Groningen, Beatrix Children’s Hospital, Groningen, the Netherlands

**Keywords:** Preschoolers, Symptoms, Healthcare professionals, Teachers

## Abstract

**Background:**

Preschoolers experience physical symptoms, like abdominal pain or minor injuries, almost every day. These experiences may shape how they deal with health issues later in life. To gain insight into these early life experiences, information from multiple perspectives is useful. This qualitative study aimed to explore important themes in preschoolers’ experience of physical symptoms, using adult professionals from various backgrounds as informants.

**Methods:**

20 semi-structured interviews were performed with professionals from different fields in healthcare and education, to learn about their ideas and observations on preschoolers’ experiences with physical symptoms. The interviews were verbatim transcribed and coded in Atlas.ti by two independent coders, after which thematic content analysis was applied to define themes.

**Results:**

Three themes emerged from the interviews: unawareness, seeking attention, and parental influence. Unawareness refers to the professionals’ idea that preschoolers have limited cognitions about causes and consequences of physical symptoms. Seeking attention was described as important for preschoolers with symptoms, both as comfort and in a social context. Professionals described diminished attention-seeking behavior in preschoolers with more severe symptoms. Parental influence was seen as highly relevant in preschoolers’ experiences with physical symptoms, with both supportive and disruptive aspects. Healthcare professionals differed from educational professionals in their observations and ideas, especially about underlying mechanisms influencing symptoms.

**Conclusions:**

Professionals report attention-seeking and parental influence as important factors in preschoolers with physical symptoms, and they report limited cognitions about causality. Professionals in healthcare and education show different perceptions, suggesting the importance of incorporating both views into research.

**Supplementary Information:**

The online version contains supplementary material available at 10.1186/s12887-024-05157-4.

## Introduction

Young children experience physical symptoms nearly every day [1, 2]. Most physical symptoms occur in everyday life and could be anything from abdominal pain to minor injuries in the playground. Besides the everyday life experiences, physical symptoms may also occur in a clinical setting, for example, when visiting a doctor or a dentist. All these experiences provide learning opportunities, with children gaining knowledge from their caregivers on how to respond to their physical symptoms via social learning [3, 4]. Eventually, early life experiences may shape how children handle illness and symptoms later in life. More insight into how young children perceive and experience physical symptoms might thus be relevant for a better understanding of the development of symptom perception and illness behavior.

For optimal insight into the development of children’s experiences and responses to symptoms, it is important to study children from a young age onwards. Previously, studies showed that four- and-five-year-old children are able to describe experiences and have ideas about their physical symptoms [5–9]. Furthermore, children at this age start attending preschool, thereby expanding their social context and social learning environment beyond their family. However, preschoolers are still in a developmental stage known as the pre-operational stage of development, meaning they are still developing meta-thinking and reflective skills [10]. When investigating this age group, it is important to consult other informants to obtain information on preschoolers’ experiences with physical symptoms. Moreover, perceptions of physical symptoms may differ between children, parents and professionals [11, 12]. To gain general knowledge on this topic, informants that encounter many different children dealing with physical symptoms are preferred. Parents, though valuable informants, are less informative since they can only report on their own children, and they can only describe their own role in such experiences from an insider’s viewpoint.

Professionals from different fields might have ideas about preschoolers’ experiences with physical symptoms, shaped by their observations and work experiences, but also based on their background and expertise. There are multiple fields of pediatric professions in which professionals encounter children with physical symptoms. However, the majority of research focuses on the clinical setting, which only accounts for a minority of the physical symptoms experienced by healthy preschoolers. Studies show that teachers frequently encounter health problems and pain incidents in their pupils [1, 2, 4, 13]. Besides regular teachers, sports teachers frequently observe painful incidents in a different setting [14]. Research on teachers’ experiences of children with physical symptoms is scarce and mainly focuses on older children or children with special needs [15, 16]. A combined study of professionals working in both healthcare and education could provide a more comprehensive understanding of preschoolers’ experiences with physical symptoms.

The main objective of this study was to explore the ideas and observations of pediatric professionals working in both healthcare and education regarding preschoolers’ experiences of physical symptoms. We performed 20 qualitative interviews with professionals working in various pediatric fields, using a phenomenological approach [17]. Our interview data underwent thematic analysis to gain a better understanding of how professionals perceive preschoolers’ experiences with physical symptoms.

## Methods

### Participants

Our research was conducted in line with the Consolidated criteria for Reporting Qualitative Research (COREQ) checklist [18]. We included 20 professionals who worked with children in healthcare or education. The key criterion for participant inclusion was their direct exposure to preschoolers in contexts where the children might experience physical symptoms. Furthermore, all participants needed to have at least one year of experience in working with preschoolers. All participants signed an informed consent form. As the current study was not clinical research with human subjects as defined in the Dutch Medical Research Involving Human Subjects Act (WMO), a formal evaluation by the ethics board was not indicated according to the Dutch law [19, 20]. 

Participants were mostly selected using purposive sampling, with a few additions using snowball sampling. The goal was to select a diverse group of Dutch-speaking participants with different backgrounds, sex, age and years of experience. The authors held brainstorm sessions to select participants from various professions. Some participants suggested additional professions that might add useful insights. Participants were approached via personal contacts or via their employer. Some professions were represented more than once but these participants differed in personal characteristics. The professionals worked in different regions of the Netherlands, both rural and urban, and within diverse populations in terms of socio-economic status. The participant characteristics are shown in Table 1.

**Table 1:** Participant characteristics

Participants	Workplace	Sex (Male/female)	Age (in years)	Parent?
**Healthcare**				
Pediatric physiotherapist 1	Physiotherapy practice	F	61	Yes
Pediatrician 1	Academic pediatric hospital	F	42	Yes
General Practitioner 1	General practice	F	50	Yes
General Practitioner 2	General practice	M	42	Yes
Psychologist	Child psychiatry department	F	35	Yes
Dentist	Academic dentistry department	M	61	Yes
Pediatric physiotherapist 2	Academic hospital	F	55	Yes
Pediatrician 2	Pediatric department regional hospital	M	60	Yes
Pediatric Dentist	Dentistry practice	F	60	Yes
Child psychologist	Child support organization	F	29	Yes
Clinical psychologist	Pediatric department regional hospital	F	64	Yes
Pediatric nurse	Pediatric department regional hospital	M	53	Yes
**Education**				
Kindergarten teacher 1	Retired	F	68	Yes
Childcare worker	Day care organization	M	26	No
Gymnastics teacher 1	Gymnastics school for toddlers	M	25	No
Gymnastics teacher 2	Gymnastics school	M	37	Yes
Tennis teacher	Tennis school	M	32	No
Ballet teacher	Dance school	F	27	No
Kindergarten teacher 2	Primary school	F	37	Yes
Kindergarten teacher 3	Primary school	F	64	No

### Interviews

The interviews were conducted individually by two interviewers, SZ, a female PhD-student and medical doctor, and DS, a male student of applied psychology, in 2019 and 2021. The first set of ten interviews was conducted face-to-face by SZ, with participants being interviewed either at their workplace or at home and the first interview serving as pilot interview. The participants were interviewed in random order regarding their professions. Due to the COVID-19 pandemic, DS conducted the second set of ten interviews online using Microsoft Teams. Most interviews lasted between 25 and 35 min. All interviews were audio recorded and then transcribed verbatim by the interviewer.

The semi-structured interview guide aimed to explore participants’ experiences and observations with children experiencing physical symptoms. Participants were asked about their observations, ideas and experiences regarding behavior, verbal expressions and emotions related to physical symptoms in preschoolers and their parents. Additionally, they were questioned on possible internal and external influences on the child’s experience of symptoms, such as the environment, parents, and stress. The interview guide was designed with a phenomenological approach, with focus on experiences and perceptions rather than exploring theories participants had [17]. Some questions were loosely based on models of symptom perception for adults, such as the Common-Sense Model by Leventhal [21]. However, the interview guide was designed to allow space for new ideas. When participants made unexpected remarks or mentioned other possible influences, some of those answers were used to add questions to the interview guide. Some changes were made based on the pilot interview, but additions happened throughout the first set of ten interviews. A translated version of the final interview guide is shown in Table 2. As the interview focused on four- and five-year-old children (preschoolers in the Netherlands), the interviewer frequently checked whether answers of participants concerned the correct age range.

**Table 2:** Interview guide (translated version)

Interview guide professionals
**General questions:**
- Age, sex, siblings, children of your own? - How much experience do you have working with preschoolers/children? - Why do you work with (young) children? - How often do you encounter children with physical symptoms? What role does it play in your work?
**Symptom experience:**
- How do you notice children suffering from (physical) symptoms? → *Behavior and change of behavior? Difference between acute and chronic? Headache/abdominal pain (internal) vs. blood/injury/bruises (external)?* → *Are chronic symptoms (Medically unexplained symptoms?) present at this age?*
- How do children talk about physical symptoms? → *Choice of words? (Specifying symptoms? Using words other than ‘pain’?) Words for symptoms*,* causes*,* does their story match their parents’ story?* → *Cause-effect*,* incorrect cause*,* recognizing symptoms from earlier on*,* fear of repeated symptoms? Consequences?*
- What emotions do children show? → *Influence of emotions on symptoms? Fear? Of what? Anger?*
- What do children do when they have physical symptoms? (behaviors) → *Solutions? Comfort? With whom?*
- How do parents respond to symptoms in their children? → *Influence on symptoms*,* influence on behavior of child*,* influence on talking?*
- How do you respond to children’s physical symptoms? → *How do they react to your reaction?* → *How do children at this age respond to symptoms of others?*
**Possible influences/other:**
- Does pretending/copying others occur at this age? - Are there other environmental factors that influence how symptoms are experienced? → *Stress*,* location*,* people around?* - Do you observe a difference between boys and girls? - Do you notice a difference in social and cultural background on how children talk about and deal with symptoms?

### Analysis

The analysis was carried out by SZ, DS and JG, trained as a child- and adolescent psychiatrist and experienced in qualitative research. JR, researcher in the field of psychosomatic medicine, and MV, pediatrician, were regularly involved in the discussion of codes and themes and in the interpretation of results.

All interviews, including the pilot interview, were coded double blind by SZ and DS, with JG supervising the coding of the first set of 10 interviews. Coding was performed using Atlas.ti. Before and during the coding of each interview, no discussion occurred between coders. Open coding was used for the first interview, after which the codes of the different coders were compared. After agreement was reached, the second transcript was coded, by using the existing codes and adding supplementary codes if necessary. After each coding session, the final encoding of the transcript and the list of codes were discussed, and codes were renamed, merged, and split. During the coding process, JG and JR were consulted regularly about the final codes. After 15 interviews, no new codes emerged and saturation was reached [22, 23]. 

After coding all interviews, the outcomes were thematically analyzed by One Sheet of Paper (OSOP) analysis by SZ and JG [24]. This is a method of visually rearranging the data from the interviews on a large piece of paper, followed by the definition of themes [24]. After coding all interviews, SZ and JG inspected the codes and associated quotations. They started with a code that had many quotations, writing these quotations on a large piece of paper and visually arranging them. Quotations from related codes were added using a different color. After completing an OSOP, they reviewed the visual arrangement to identify connections. The results were then discussed with the whole research team to define themes. This process continued until all relevant codes were covered and the defined themes aligned well with the overall interview data. The coding and analyzing process was logged by the researcher to keep track of the thinking process and decisions that were made [25]. The text of the draft paper was shared with all participants.

## Results

### General findings

All participants shared ideas and observations about their experiences with preschoolers having physical symptoms, both answering questions and spontaneously. There were no substantive differences between the interviews conducted in 2019 and the interviews conducted in 2021. We observed differences between healthcare and educational professionals. Healthcare professionals seemed to talk a lot more about the parental influence on (dealing with) symptoms. Furthermore, they mentioned more ideas and theories on underlying mechanisms, such as stress, developmental disorders, family dysfunction or socio-cultural influences. A few healthcare professionals also spoke of children’s resistance and fear towards them. Educational professionals seemed to talk more about children’s behaviors in the context of symptoms, such as seeking attention, crying, or withdrawing. They also seemed to speak more about their own appraisal of whether the physical symptoms of children were real and the possibility of children pretending to have symptoms. The necessity of experience and personally knowing a child was frequently mentioned as important to interpret behaviors related to symptoms. Furthermore, the educational professionals provided observations on group dynamics following the experience of physical symptoms in one of the pupils.

Besides these differences, the information given by participants generally showed many similarities, which led to three main themes concerning preschoolers and physical symptoms. These can be broadly categorized as being related to cognitions, behaviors and social interactions.

### Cognitions: unawareness

The first theme that emerged from the interviews was unawareness. Many participants expressed preschoolers to be too young to understand their experiences of physical symptoms properly. This theme concerning the cognitions of preschoolers can be split into causes of physical symptoms and consequences of physical symptoms.

(General practitioner 1)

“*Interviewer: … Are they talking about the symptom itself? Or about its cause?*

*Participant: Well, at this age not about causes. No.*

*Interviewer: And about the consequences?*

*Participant: Well also not, no. In my experience, it’s pretty simple at this age: I have pain here.*”

All participants were asked about preschoolers’ cognitions about their physical symptoms. Most participants indicated that they have little understanding of their symptoms unless the cause is obvious, for instance if they have a small injury from falling on their knee. With visible injuries, some participants stated that preschoolers can describe the cause, and sometimes give detailed reports.

(Pediatric nurse)

“*Yeah, yeah, yeah. That’s when the child can really indicate: ‘gosh, I have pain there and this is how it happened.’ To the point of a broken arm, for example. Yeah, then they know they fell and all they do is support that arm.*”

In the absence of a clear injury, multiple participants indicated that preschoolers generally do not seem to have ideas about the causes of their physical symptoms. Some participants mentioned that if preschoolers spoke to them about the causes of their symptoms, these causes were clearly suggested by their parents.

(Pediatric physiotherapist 2)

“*Yes, I think so, but that might also depend on how the parents informed the child, because of course if the parents mentioned often enough that it’s because you fell, then eventually that becomes a truth. When you’re four, five years old.*”

One participant, a pediatrician, mentioned that some preschoolers with chronic conditions are able to link their behaviors to their physical symptoms.

(Pediatrician 2)

“*Yes, yes, there are some children who can explain that ‘whenever I do this, whenever I have to be there, I have this.’ Yes.*”

When asked about preschoolers’ cognitions of consequences of physical symptoms, some participants mentioned that they could not answer that question or that preschoolers do not have cognitions about the consequences yet. Others indicated that preschoolers do have cognitions about consequences, sometimes simple (e.g., injuries need Band-Aids), and sometimes more advanced. These more advanced cognitions can be seen in their descriptions of preschoolers pretending to have symptoms.

(Psychologist)

“*Actually yes, sometimes. Because of course there are also children who say they have a tummy ache so they won’t have to go to school.*”

Pretending was often mentioned as a way of getting attention, but also to avoid something they do not want to do (e.g., going to school, doing a difficult exercise), to get treats or as a way of learning social interactions. Most of the examples of preschoolers pretending to have symptoms were perceived as innocent and participants described the pretending as normal behavior for this age group. However, a few participants indicated that they interpreted pretense symptoms as a sign of underlying stress or difficulties in the family context.

While the majority of participants had experience with preschoolers pretending to have symptoms, some participants, all working in clinical professions, indicated that preschoolers are too young to feign symptoms.

(General practitioner 2)

“*But children don’t pretend. Preschoolers don’t pretend. Them pretending is something I don’t really believe in.*”

Many participants agreed that the unawareness of children starts to diminish at preschool age and mentioned that older children have more cognitions and reactions that are more complex.

### Behavior: seeking attention

The second theme that emerged from the interviews was seeking attention. Professionals expressed preschoolers to seek attention when having physical symptoms for several reasons. Many participants mentioned preschoolers with physical symptoms to seek attention for safety and comfort from a familiar person, usually a parent or teacher. A few participants described that this may ease the physical symptoms or takes them away.

(Ballet teacher)

“*… Yes, with tummy aches that’s often the case, if they know it themselves, when they’ve shared that they have a tummy ache, that it’s already, that the pain is already less.*”

Seeking attention was also mentioned as a way of being acknowledged by the professional or by peers. Participants, mainly working in education, described preschoolers in a group using or exaggerating a physical symptom as a way of achieving attention from the professional, regardless of it being a real or a pretended symptom.

(Kindergarten teacher 2)

“*… also examining their body in terms of ‘do I have blood anywhere?’ A preschooler could even, if they have a very small wound, start squeezing their finger very hard (squeezes own finger as an example) to make a drop of blood come out.*”

(Gymnastics teacher 1)

“*Interviewer: Okay. And do you ever see kids pretending to be suffering from something?*

*Participant: Yes, of course, maybe even more of those. Yeah, no, you also have children who kind of use pain as attention of course. Who don’t have pain.*”

A few educational participants described the preschoolers in their classes copying each other’s symptoms, as a way of gaining attention. These participants viewed these processes as part of the social learning of children this age.

(Kindergarten teacher 2)

“*Uhm, but they also see when one of them says ‘Oh I hurt my finger, it hurts’, the teacher then says ‘Oh dear, what happened?’ And that is attention, right? (…) And that’s what the other one wants too, they also want attention from the teacher and to show the desirable behavior.*”

Some participants mentioned the social context as an important factor in the seeking of attention. They described observations of how preschoolers with physical symptoms appear to adjust their reaction to the symptom based on whether a familiar person is available to provide attention.

(Kindergarten teacher 2)

“*Well, it varies a lot, very often preschoolers will look around like: ‘did anyone see that I fell over?’ And if someone did see it, they will start crying, uhm, because it’s also a little bit like: oh right, that’s the way these things go. But very often they look around and go ‘oh no one has seen me’, then they get up and carry on.*”

Professionals described the behavior of seeking attention mostly in the context of physical symptoms that were milder or pretended. They described preschoolers not to seek attention when experiencing a more serious physical symptom and shared observations of children turning quiet, sitting still or pulling back from playing. This quieter, withdrawing behavior was often reported with physical symptoms that were more severe or “real.”

(Gymnastics teacher 2)

“*… So, if children are crying and standing up, or lying down and crying, then it’s really a sign that things are going well. If they stay still, and um, so um you don’t hear them, then often something really serious is going on.*”

Moreover, this quieter behavior was also mentioned in the context of crying behavior and symptoms. Professionals often raised that they felt the crying behavior did not match the severity of the physical symptom, with crying loudly observed in less severe symptoms and no crying or crying quietly observed in more severe situations.

(General practitioner 1)

“*Well, look if it’s something serious, you see that when children are in a lot of pain, um, they’re usually actually not very loud. So, many children who are really sick, or who have really fractured something, who, yes maybe if you’ve just had a fracture, you cry a lot, but once they come to me the children who are really suffering from something are often remarkably quiet.*”

### Social interactions: parental influence

The third theme that emerged from the interviews was parental influence. Participants mentioned their views on parents and parental influences many times during the interviews, both answering questions and spontaneously. Within the theme of parental influence, participants described both supportive aspects and disruptive aspects. When expressing supportive parental behaviors, many participants talked about how preschoolers need their parents in the case of a physical symptom.

(Gymnastics teacher 2)

“*Well, the first emotion is ‘mommy’, yeah, they want to go to mommy right away. If something really severe happened, then it’s mommy. Yeah. Um, then I will always say, ‘go sit on the bench’, if something really bad happened, and ‘I will call your mommy and your mommy will come and pick you up soon.’*”

Participants described how parents provide the child with safety, calmness, and reassurance. Furthermore, participants spoke about parents setting an example of how to react, with their children mirroring their reactions. This reaction could be calming and normalizing, or providing behavior regulation.

(Dentist)

“*Very important is the, as I was saying, is how the parents themselves deal with it. If the parents are very relaxed about it, the child often comes in relaxed as well. And if the parents are stressed, then the child is also stressed. It’s really a projection of the parents’ experience and the parents’ expectations that determine how a child experiences it.*”

Another supportive aspect was the signaling function parents were reported to have when their children experience symptoms. Parents were reported to assess the severity of the symptoms of their children, to interpret their children’s behaviors concerning physical symptoms, and to decide whether a healthcare professional is needed. In their work, many participants often need the parents as a source of information concerning the physical symptoms of their child.

(General practitioner 1)

“*Interviewer: But can they indicate that, often, like, it hurts right here?*

*Participant: No, very often they can’t. Very often you won’t get a response. So, then you do have to hear it from the parents, at that age.*

*Interviewer: Okay.*

*Participant: At that age, it’s still very difficult.*”

Some participants described the parents as the primary source of information when preschoolers are incapable of giving information or to check the information given by preschoolers. Other participants see parents as a secondary source of information, complementary to the information received from preschoolers or their own observations.

However, professionals also described some aspects of parental influence to be of a more disruptive nature. Some participants mentioned the influence of parental emotions, such as panic, fear or being overly concerned to have a negative effect on their children’s stress and/or symptoms. Others interpreted a lack of supportive behaviors, or parents ignoring their child as more disruptive aspects.

(Gymnastics teacher 1)

“*Well, let me put it this way: if I do nothing, or if a parent does nothing, then that child will get more and more upset. Um, so in any case they need a parent to be calm and offer comfort. And it’s not gonna be good enough if the parent is panicky. That, that’s not, then nothing will happen.*”

A few participants brought up their need to verify the parents’ assessment of their child’s symptoms, as parental assessment might be colored by their emotions. Participants sometimes reported to be hindered in their work by disruptive parental behaviors.

(Pediatric physiotherapist 1)

“*Yeah, there’s always a parent present. Right? And so, to what extent can you disconnect them from that. Sometimes that is very tricky indeed. Yeah. At this age, you simply have the parents there. And then you just deal with it as it is.*”

## Discussion

Exploring the perspectives of professionals on preschoolers dealing with physical symptoms, we identified three themes. Firstly, professionals generally described preschoolers as unaware when it came to physical symptoms, particularly concerning the causes of their symptoms. Secondly, professionals reported the importance of attention seeking behavior. Preschoolers were reported to seek attention for comfort, but also to be acknowledged in a social context. Seeking attention was described in the context of milder physical symptoms, with professionals observing withdrawing behaviors in preschoolers with more severe symptoms. Thirdly, parents were reported to play an important role in their children’s experiences with physical symptoms. Professionals described parents’ supportive influences such as providing comfort, and disruptive influences of parents being scared, panicky or ignoring their child. Professionals also described parents as a source of information.

The first theme we identified was unawareness. Participants in our study reported preschoolers to have little knowledge on causes of physical symptoms. However, research shows that preschoolers are able to make statements about the causality of their physical symptoms [5, 8, 26–28]. Therefore, our participants seemed to underestimate cognitions of preschoolers, which is in line with earlier research in pediatric professionals and students from pediatric professions [29, 30]. One possible explanation for this underestimation could be too little or suboptimal conversation about cognitions between professionals and preschoolers. After all, if professionals presume preschoolers not to think about causes, they might be less likely to start a conversation about it.

The second theme that emerged in our study was seeking attention. Attention seeking when experiencing physical symptoms has been described in the context of parental attention to children’s everyday pain [31]. In line with our results, parents described their young children to modify their pain reactions for different purposes such as gaining attention or avoiding unpleasant activities. Furthermore, interviews with preschoolers show that they are aware that one can feign a physical symptom to achieve something [8]. We found that professionals in both healthcare and education observed attention-seeking behaviors to be less prominent or even absent with more severe physical symptoms. Our results on this withdrawal behavior showed similarities with research on sickness behavior in young children, although sickness behavior was seen in the context of infection rather than pain or other physical symptoms [32]. Withdrawal behavior, which the professionals in our study often mentioned in the context of more severe physical symptoms, has been described as a cue of pain for parents [33, 34]. Furthermore, crying was mentioned in association with less severe physical symptoms, which seems contrasting to existing literature. Interestingly, when asking preschoolers about crying and physical symptoms, they see crying as a signal of severe physical symptoms [8]. Furthermore, most pain assessment scales link the intensity of crying to the level of pain [35]. A possible explanation for the discrepancy we found with the existing literature on severity of crying, may be that studies are usually focused on ill children, compared to our focus on everyday symptoms.

The third theme we identified was parental influence. Unsurprisingly, our study shows the importance of parents concerning physical symptom experiences in preschoolers. A supportive and comforting role of parents was also described as important by preschoolers themselves, both in everyday and clinical settings [6, 8, 36]. Furthermore, this result is consistent with the attachment theory in which distress or pain activates attachment behavior with the child seeking proximity and security from a caregiver [37, 38]. In research on procedural pain, such comforting reassuring behavior is interpreted as distress-promoting with distraction being coping-promoting [39, 40]. While distraction might be more important during the predictable symptoms caused by medical procedures, comforting behavior might be just as relevant after the procedures and during unpredictable everyday symptoms. However, research on parental influences in the everyday setting remains limited [4]. Consistent with the Social Learning theory, participants indicated that parents are an important example for their preschool-aged children, which was also described in a recent Delphi study with a mixed sample of healthcare and educational professionals [3, 41]. 

To the best of our knowledge, this is the first interview study on physical symptoms in preschoolers combining the perspectives of different professionals in healthcare and education. Other research focused on the different perceptions of healthcare professionals and teachers on symptoms in the context of a specific disease or subgroup of children [42, 43]. The differences we found between healthcare professionals and educational professionals can mostly be explained by the different settings in which they encounter and observe preschoolers with physical symptoms. Healthcare professionals often have brief encounters with children, who are in most cases accompanied by their parents. This may explain why healthcare professionals referred to experiences with parents more frequently than educational professionals, who often see children without their parents. Another difference was the tendency of educational professionals to speak more on behaviors compared to the healthcare professionals, who, despite the phenomenological design of the interview guide, shared more theories on underlying mechanisms and conditions. This difference may again be explained by the different duration of contact with the preschoolers, with teachers observing children for a longer time and building a relationship. Comparatively, healthcare professionals often have a relatively superficial relationship with the children and may rely more on theory as they have less time to observe. Another possible explanation may be the difference in training, with more academically trained participants in the group of healthcare professionals, compared to the education group.

Our study has several strengths and limitations that need to be emphasized when interpreting the results. A strong aspect of this study is its thoroughness in methodology and documentation. Our study was conducted in line with the COREQ checklist [18]. For insight in our methodology and documentation, we uploaded our logbook and interview guide to the website of the Open Science Framework [25]. The study’s strength lies in its diverse sample, encompassing various pediatric professions from both healthcare and education. We achieved variation in participant sex, age, experience, and profession, as well as in the populations of children they worked with, contributing to a comprehensive overview. Lastly, this study was conducted in a multidisciplinary team with different levels of experience and backgrounds, both clinical and non-clinical. The differences between the two interviewers may have resulted in different interview styles, which may have enriched the collected data. As the analysis was conducted in a team consisting of expertise in pediatrics, psychiatry, psychology and psychosomatic medicine, we were able to construct an interdisciplinary perspective.

This study also has limitations worth noting. As our participants have mostly been selected from the network of the researchers, there might be a possible selection bias. We tried to avoid this by also approaching participants via their employer. Another possible limitation is the cultural background of our participants. While our sample varied on many factors, little cultural diversity existed in the sample. Participants from varied cultural backgrounds might have enriched the data.

More research is needed on children’s experiences of physical symptoms, as these may be influential throughout life. As parents play an important role in their children’s symptom experiences, further research is needed to chart the parents’ perspective and role, preferably in a quantitative, longitudinal design. With observations and questionnaires, the behaviors and cognitions of parents and their influences on the symptom experiences of their children may be investigated on a larger scale. Specifically, it may be interesting to further explore parent’s thoughts on their children’s feigning of physical symptoms, and their ideas on possible reasons for their children to do so. This phenomenon has barely been reported in literature so far. Furthermore, it may be interesting to investigate the discrepancy we found with the literature regarding intensity of crying and severity of symptoms, to explore whether parents also observe an inverse relation between these factors.

The current study has clinical implications as well. Our results showed differences between healthcare and educational professionals working with preschoolers, suggesting that interactions between clinical and non-clinical professionals could enhance the knowledge of both groups. Healthcare professionals might gain better understanding of preschoolers when they are trained together with educational professionals who have more behavioral observations. Additionally, all professionals working with children, whether they work in healthcare or in education, may benefit from education about communication and physical symptoms. Professionals should learn to address physical symptoms from a biopsychosocial perspective, as they play an essential role in responding to children’s symptoms, and helping children by learning them to regulate their symptoms. Training opportunities could include learning about effective (co-regulating) communication about symptoms, using positive language.

Our results also suggest a clinical implication regarding the potential underestimation of preschoolers’ cognitions about causes of physical symptoms. Healthcare and educational professionals should be aware of this underestimation and attempt to improve conversations with preschoolers about their ideas regarding their symptoms. In medical practice, involving children in consultations could enhance the connection between professionals and the child’s perspective.

## Conclusions

Our study showed that preschoolers’ experiences of physical symptoms are multifaceted according to professionals from healthcare and education. Professionals report on preschoolers’ limited cognitions about causality of physical symptoms, and their attention-seeking behaviors in the context of physical symptoms. Parental influence is described as an important factor in preschoolers dealing with physical symptoms, with both supportive and disruptive aspects. The varied professional perspectives underscore the need for comprehensive research and training, integrating both healthcare and education perspectives.

## Electronic supplementary material

Below is the link to the electronic supplementary material.

Supplementary Material 1

## Data Availability

The datasets used and/or analyzed during the current study are available from the corresponding author on reasonable request.
